# Supplementary material for: Identification of 3′ UTR motifs required for mRNA localization to myelin sheaths in vivo
Source: PLoS Biol. 2021 Jan 13;19(1):e3001053. doi: 10.1371/journal.pbio.3001053 (PMC7837478; doi:10.1371/journal.pbio.3001053)
Supplement: Attachment — Submitted filename: ReviewAppel PLoSBiol2020.docx [file pbio.3001053.s019.docx]

Localised mRNAS and local translation into protein is increasingly recognized as a guiding principle in many biological systems. In particular neurons utilize this to localize and locally translate many mRNAS for example, at synapses. It is increasingly recognized that this principle is used by oligodendrocytes to sort mRNAs into forming myelin and indeed *mbp* mRNA was discovered early on to be a pertinent example here. The present paper is investigating the identity of 3´UTR motifs required to localize the abundant number of mRNAs found in myelin. They utilise the power of living zebra fish combined with the MS2 system developed by the lab of Robert Singer for tagging mRNAs, quantifying and visualizing their subcellular localisation after attachment to a fluorescent EGFP reporter binding the MS2 loops. Their EGFP reporter is driven by a sox10 promoter to allow expression in oligodendrocytes and has an NLS sequence added to reduce non-specific fluorescence.

The authors initially tested their tools using the well-characterised 3´UTR of *mbp* mRNA coupled to a Scarlet-Caax reporter which is translated into protein. As a control they utilized the 3´UTR of SV40. This also allowed them to define the optimal time window (4dpf) for visualising endogenous *mbp* mRNA using single molecule FISH. They observed that this co-localised with a F-actin reporter indicating that *mbp* mRNA occupies the leading edge of the forming myelin sheath. In contrast, the control *sv40* 3´UTR did not enrich at the leading edge.

They subsequently investigated the published data of mRNAs localised in murine myelin from P18 brains. The myelin was obtained using a classical biochemical purification of myelin over density gradients, which does not exclude a degree of contamination, for example, with cell processes. They selected candidate 3´UTRs using the GO terms oligodendrocyte, myelin, translation and synapse, also looking for zebra fish othologs. They came up with six candidate genes: *cadm1b, cyfip1, dlg1, eif4ebp2, fmr1* and *lrrtm1.*

Cadm1 and Irrtm1 are both transmembrane proteins which would be synthesised on rough ER. In fact, their latest very interesting Nat. Comm. paper has examined Cadm1 in detail. On the whole, it is mRNAs which are translated on free ribosomes that exhibit long 3´UTRs directing localization and local translation in cellular compartments. *It is thus not surprising that the 3´UTRs of Cadm1 mRNA does not promote localisation to myelin. I would recommend that this point should be brought out and discussed more clearly in the Discussion*. In fact, I would have selected initially other candidates that coded for proteins translated on free ribosomes instead of these two genes, although they clearly serve as good negative controls. Interestingly though, *Irrtm1* 3´UTR did localise to myelin sheaths! *Perhaps they could discuss this point further.*

They succeeded in identifying three new motifs in the 3´UTRs of three (*mbp, eif4ebp2* and *fmr1*) of the localised mRNAs which contain cis-regulatory motifs. These are absent fromn the non-localised mRNAs and also not present in *Irrtm1* 3`UTR. One of these motifs is found abundantly in the myelin transcriptome.

This is thus an important paper, using state of the art technology as it demonstrates *in vivo* sorting principles of mRNAs to myelin and has identified new motifs playing a role here.
