# Supplementary material for: Identification of 3′ UTR motifs required for mRNA localization to myelin sheaths in vivo
Source: PLoS Biol. 2021 Jan 13;19(1):e3001053. doi: 10.1371/journal.pbio.3001053 (PMC7837478; doi:10.1371/journal.pbio.3001053)
Supplement: Attachment — Submitted filename: Reviewer_Response_v2.docx [file pbio.3001053.s021.docx]

Thank you for the opportunity to revise our manuscript. As detailed below, we performed an extensive set of additional experiments as requested by Reviewer 3. The results of testing RNA localization mediated by the individual sequence motifs is not straightforward. We believe that this reflects the importance of sequence context, which could be determined, for example, by RNA secondary structure or RNA modification. Such context-dependent effects have precedent and we cite examples from the literature.

Reviewer #3: In my previous review, I had raised major and minor points relating to the conclusions that can be drawn from the data as they are presented. These concerns remain because the authors do not provide additional data to address my questions.

1. I asked for (at least correlative) evidence that differences in mRNA abundance correlate with the properties of the respective myelin sheath (e.g. length). This point has not been addressed in the revised version so that the reader is left with notion that distal mRNA transport is crucial for local regulation of myelin growth (see introduction), but no evidence is provided that this is actually the case in the system presented here.

Response. We measured endogenous *mbpa* transcript density within individual myelin sheaths, revealed by smFISH, and compared these data to sheath length. The data are shown in Figure 2E. We made the following addition to the text, beginning on line 154: “At 4 dpf, the density of *mbpa* mRNA and sheath length were positively correlated indicating that longer sheaths have more *mbpa* mRNA (Figure 2E). We interpret these data to mean that longer nascent sheaths have more cytoplasmic space and therefore have a greater capacity to accumulate macromolecules such as mRNA. In support of this interpretation, previous reports have found that longer myelin sheaths have increased mitochondrial densities(38).”

2. I had asked for additional experimental information about the necessity and sufficiency about motifs 2 and 3, because although being present in many distally transported transcripts, only the motifs derived from the mbp 3'UTR seem sufficient for distal mRNA targeting. The authors now discuss possible explanations for this circumstance, which may all be true. However, the significance of the consensus motifs remains very difficult to assess.

Response. To determine why motif 2 and/or 3 are sufficient in only one context (*mbpa* 3’ UTR), the original suggestion was to exchange motifs between the candidate 3’ UTRs. However, this approach would require 84 possible motif combinations to fully test the question. As an alternative, we used a reductionist approach, similar to our experimental design in Figure 6, to test the sufficiency of each motif individually. Specifically, we isolated a total of 8 sequences from three 3’ UTRs and used the MS2 system to determine which sequences are sufficient for mRNA localization to myelin sheaths. The new data are presented in Figure 7E and we added the following passage to the Results beginning on line 295: “To test the sufficiency of individual sequence motifs, we inserted each sequence flanked by 6-10 nucleotides on each side upstream of the *sv40* 3’ UTR. We found that 4 out of the 8 sequences, including *mbpa* motif 3, *fmr1* motif 3, *eif4ebp2* motif 1, and *eif4ebp2* motif 2, were sufficient to localize the reporter mRNA to nascent sheaths. The variability in our data suggests the motifs are sensitive to contextual features intrinsic to the cis-elements. For instance, primary sequences (52) and secondary structures (53) frequently modulate RNA regulatory mechanisms (54,55). Here, we find that the sufficiency of each motif to drive mRNA localization to myelin is context-dependent. For instance, *eif4ebp2* motif 1 and 2 are sufficient for mRNA localization independently (Figure 7E); however, in the context of motif 3 mRNA localization is repressed (Figure 7D). Furthermore, when *mbpa* motif 2 and 3 are coupled, they are sufficient for localization (Figure 7D). However, when tested independently, we found that motif 3 accounts for the majority of the mRNA localization (Figure 7E), indicating that motif 2 does not repress mRNA localization (Figure 7D). Overall, these data highlight the complexities of mRNA localization and add to the growing body of evidence that interactions between cis-regulatory elements and trans-acting factor are context-dependent (52–57).”
