# Supplementary material for: Identification of 3′ UTR motifs required for mRNA localization to myelin sheaths in vivo
Source: PLoS Biol. 2021 Jan 13;19(1):e3001053. doi: 10.1371/journal.pbio.3001053 (PMC7837478; doi:10.1371/journal.pbio.3001053)
Supplement: Attachment — Submitted filename: Yergert&Appel_final_response.docx [file pbio.3001053.s022.docx]

No response to reviewers was requested for this revised version.
